# Opioid Use Disorder Treatment Linkage at Strategic Touchpoints Using Buprenorphine (OUTLAST-B): Rationale, Design, and Evolution of a Randomized Controlled Trial

**DOI:** 10.20900/jpbs.20230010

**Published:** 2023-12-25

**Authors:** Courtney D. Nordeck, Anjalee Sharma, Mishka Terplan, Kristi Dusek, Elizabeth Gilliams, Jan Gryczynski

**Affiliations:** 1 Friends Research Institute, Baltimore, MD 21201, USA; 2 Behavioral Pharmacology Research Unit, Johns Hopkins School of Medicine, Baltimore, MD 21224, USA; 3 Baltimore City Health Department, Baltimore, MD 21202, USA

**Keywords:** opioid use disorder, buprenorphine, patient navigation

## Abstract

**Background:**

Despite the effectiveness and growing availability of treatment for opioid use disorder (OUD) with buprenorphine, many people with OUD do not access treatment services. This article describes the rationale, methodological design, evolution, and progress of an ongoing clinical trial of treatment linkage strategies for people with untreated OUD.

**Methods:**

The study, titled *Opioid Use Disorder Treatment Linkage at Strategic Touchpoints using Buprenorphine (OUTLAST-B)*, uses “strategic touchpoints”, initially sexual health clinics and subsequently broadened to other service venues and participant social networks, for recruitment and screening. Adults with untreated OUD (target *N* = 360) are randomized to one of the three arms: Usual Care (UC, enhanced with overdose education and naloxone distribution), Patient Navigation (PN), or Patient Navigation with an immediate short-term bridge prescription for buprenorphine (PN + BUP). In the PN and PN + BUP arms, the Patient Navigator works with participants for 2 months to facilitate treatment entry and early retention, resolve barriers (e.g., ID cards, transportation), and provide motivational support.

**Results:**

The primary outcome is OUD treatment entry within 30 days of enrollment. Participants are assessed at baseline and followed at 3- and 6-months post-enrollment on measures of healthcare utilization, substance use, and general functioning. Challenges and recruitment adaptations pursuant to the COVID-19 pandemic are discussed.

**Conclusions:**

This study could provide insights on how to reach people with untreated OUD and link them to care through non-traditional routes.

**Trial Registration:**

The study is registered at ClinicalTrials.gov (NCT04991974).

## INTRODUCTION

Opioid use disorder (OUD) is a widespread substance use disorder (SUD) in the United States (US), affecting an estimated 6–7 million adults and adolescents [1]. Opioids are responsible for the majority of drug overdose deaths in the US, which exceed 100,000 deaths annually [2], particularly as the illicit opioid supply has become dominated by highly potent fentanyl. Medications to treat OUD (MOUD), such as buprenorphine, are a proven treatment option for OUD and reduce overdose mortality [3-5], hospitalizations [6], emergency department utilization [6,7], incarceration [8], and illicit drug use [9]. Despite effective treatment options, many individuals with OUD do not seek or receive treatment services, including MOUD [10].

One approach to address this treatment gap is to use other novel service settings as initial access points for identifying people with OUD and linking them to treatment. For example, hospital emergency departments have successfully been used to initiate buprenorphine. Sexual health clinics are another service setting that may hold promise as an access point for OUD treatment. Preliminary work by our team found relatively high prevalence of OUD and feasibility of treatment linkage services in sexual health clinics [11]. Sexual health clinics are often operated by municipal health departments and see a high volume of patients, some of whom may not otherwise seek care at hospitals, SUD treatment programs, or primary care. Thus, the sexual health clinic was considered a potentially untapped touchpoint through which to engage individuals with untreated OUD in care, leveraging the opportunity of their seeking sexual health care.

### Promising Interventions for Individuals with Untreated OUD

#### Coupling patient navigation and motivational interventions

Patient Navigation (PN) is a form of strength-based case management that strategically guides individuals through the complexities of the existing healthcare system by identifying barriers to treatment entry and promotes adherence to routine healthcare. This technique is patient-centered and provides a tailored, one-on-one approach to address barriers and to facilitate community-based service utilization. PN has previously been found to improve cancer screening and follow-up rates [12], as well as entry, adherence, and viral load outcomes in HIV treatment [13,14]. More recently, PN was found to improve treatment linkage and reduce hospital readmissions among hospitalized patients with SUDs [15]. Prior studies, including randomized controlled trials (RCTs), found that approaches such as patient navigation and outreach case management can increase rates of entry to SUD treatment [16-21]. However, not all studies have shown the effectiveness of PN, such as in one large trial that found PN did not impact viral suppression among hospitalized patients with HIV and substance use at 12-month follow-up [22].

Prior research has also demonstrated that motivational interventions may be associated with reduced substance use. A Cochrane Review of 93 studies reported that motivational interventions may reduce substance use compared to no treatment in the short-term [23]. Other studies demonstrated that motivational interventions can improve rates of entry to substance use treatment among treatment-seeking and non-treatment seeking populations and are associated with reducing hospital admissions [15,16,18,24-28]. In recent years, there has been growing interest in using peers with lived experience to deliver recovery support and treatment linkage interventions–functions that have some overlap with Patient Navigation [29,30].

While there have been mixed results of the efficacy of PN and related approaches in different care settings, coupling PN and motivational interventions delivered by staff with lived experience may be a useful strategy to assist patients in maintaining continuity of care by promoting basic needs while initiating community-based treatment. Extant research and intervention evaluation has identify two types of barriers to accessing medical and SUD treatment services among individuals with SUDs: (1) internal factors, which may be augmented by SUD pathology (e.g., low problem recognition, ambivalence, fluctuating motivation, disorganization), and (2) external barriers (e.g., transportation, health insurance, treatment admission requirements such as identification cards and proof of residence). It is critical to resolve both internal and external barriers to promote engagement in appropriate medical and substance use treatment services.

#### Buprenorphine initiation at points of need

Research in other medical settings supports the practice of point-of-need buprenorphine initiation as a bridge to traditional community-based OUD treatment. Specifically, studies conducted in emergency departments (EDs) show that ED-initiated buprenorphine was associated with increased rates of entry into community-based buprenorphine treatment [31-33]. Furthermore, similar findings have been demonstrated for initiating buprenorphine during inpatient hospitalization showing that hospital-based buprenorphine initiation is associated with decreased overdose risk and increased community-based treatment engagement [34-36]. This research suggests that episodic care may be a viable opportunity to initiate MOUD and subsequently improve overall health outcomes, even if patients are not explicitly seeking that type of care. While sexual health clinics may be an episodic care setting, it is a unique opportunity to link individuals with untreated OUD to treatment services.

### The OUTLAST-B Study

This article describes the protocol, evolution, and ongoing progress of a randomized trial of treatment linkage strategies for people with untreated OUD. Originally envisioned to target sexual health clinic patients, this study was initially titled *Opioid Use Disorder Treatment Linkage at STD Clinics using Buprenorphine (OUTLAST-B)*. Subsequently, recruitment was broadened, and the title was updated from STD Clinics to *Opioid Use Disorder Treatment Linkage at Strategic Touchpoints using Buprenorphine*, retaining the same acronym (OUTLAST-B). The study seeks to test whether support from a Patient Navigator would facilitate treatment entry for individuals with untreated OUD, and whether rapid access to buprenorphine medication through a bridge prescription would further increase this effect. Participants with untreated OUD are randomized to one of three arms: Usual Care (UC) enhanced with overdose prevention resources (i.e., naloxone), Patient Navigation (PN), or Patient Navigation + Buprenorphine Initiation (PN + BUP).

### COVID-19 Related Recruitment Challenges and Novel Adaptation

**Figure 1.**
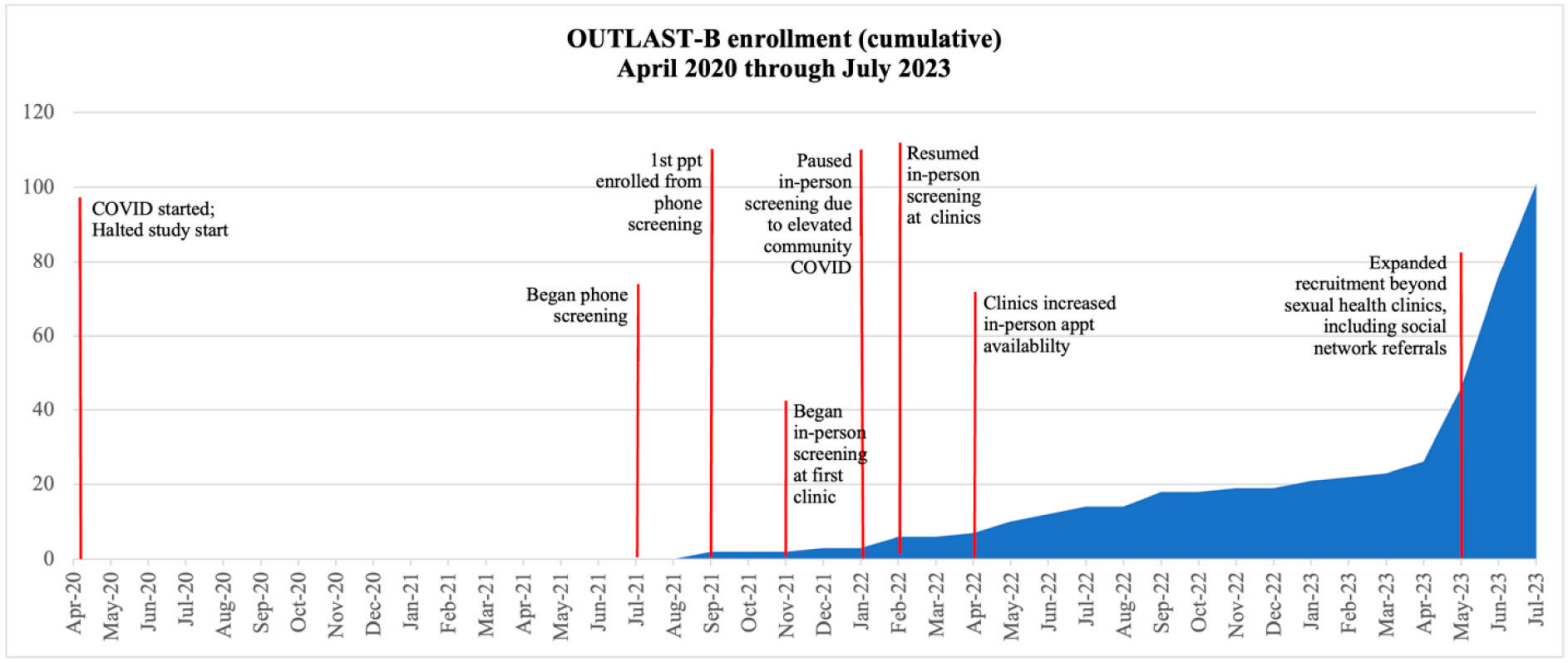
Cumulative enrollment and study challenges from April 2020 through July 2023.

The initial population for this study consisted of sexual health patients with untreated OUD. In a pilot study that took place between 2012 and 2015, approximately 11% of screened patients at the two urban, public sexual health clinic sites met diagnostic criteria for OUD [11]. This preliminary data informed feasibility decisions related to recruitment within these settings. However, due to the COVID-19 pandemic, the sexual health clinics restricted service delivery to scheduled appointments, ceased walk-in appointment availability, and primarily conducted visits through telehealth. As a result, planned in-person screening and recruitment were delayed. After approval of COVID-19 related protocol modifications, remote phone screening with sexual health clinic patients started in July 2021. A timeline of ongoing study-related challenges and cumulative recruitment is presented in Figure 1.

In-person screening at the sexual health clinics began in November 2021. However, from July 2021 through April 2023, recruitment was slow, garnering only 28 study participants. It appears that the COVID-19 pandemic not only disrupted operations at the clinics and delayed in-person recruitment, but also changed the patient population accessing care at these sites. Hence, rates of untreated OUD in the clinics’ patient population were much lower than anticipated based on prior work.

Acknowledging ongoing challenges that hindered planned recruitment efforts, the research team consulted with health department collaborators, the National Institute on Drug Abuse (NIDA), and the Data and Safety Monitoring Board (DSMB) to propose novel strategies for recruitment expansion beyond the sexual health clinic (more details provided below; see Recruitment and Progress to Date sections). Since expanding the study’s target population beyond the sexual health clinic and pursuing other strategic touchpoints, we have seen a significant increase in enrollment, particularly due to social network referrals (i.e., inviting friends, family members, and peers for screening). Despite these changes, the overarching purpose of the OUTLAST-B study is aligned with its original intent and aims to examine the effectiveness of OUD treatment linkage strategies for those with OUD and who are not actively seeking OUD treatment using patient navigation and buprenorphine bridge services.

## METHODS

### IRB Approvals and Data and Safety Monitoring

The Western Institutional Review Board/Western Copernicus Group (WIRB/WCG) IRB approved the study and provides oversight (Protocol ID: 20190610; initial approval was obtained 2019-03-20). The study is registered at ClinicalTrials.gov (NCT04991974). A federal Certificate of Confidentiality was automatically issued as part of the NIH grant award. The study is monitored by an independent DSMB.

### Study Design

**Figure 2.**
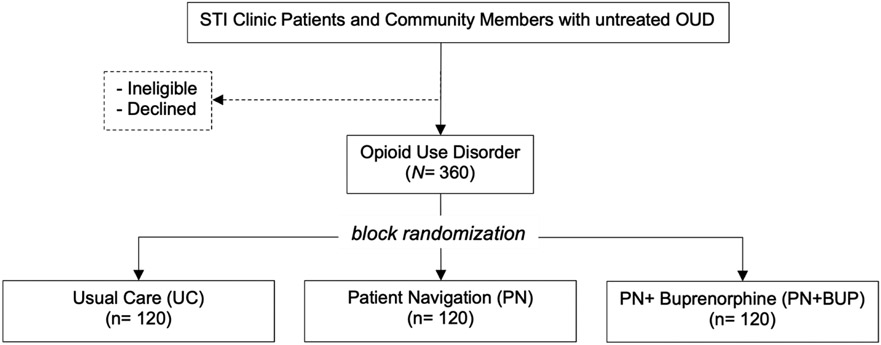
Study and Recruitment Flow.

This study will examine the effectiveness of OUD treatment linkage strategies for individuals with untreated OUD. The study is a parallel, three-arm RCT comparing Patient Navigation + Buprenorphine Initiation (PN + BUP), Patient Navigation (PN), and Usual Care (UC) enhanced with overdose prevention resources including naloxone (see Figure 2).

### Study Aims and Hypotheses

The trial will compare the study arms on three broad categories of outcomes.

*Aim 1* of the study is to determine the effectiveness of PN + BUP vs PN vs UC in facilitating OUD treatment entry (primary outcome) and retention in treatment.

*Aim 2* is to determine the effectiveness of PN + BUP vs PN vs UC in reducing opioid use, drug-related problems, and overdose events (fatal and non-fatal).

*Aim 3* of the study is to determine the effectiveness of PN + BUP vs PN vs UC in reducing incidence of HIV/STIs, increasing adherence to recommended sexual health care, and reducing HIV/STI related risk behaviors.

Our overarching hypothesis is that the PN + BUP arm will be superior to the PN only arm, which will be superior to UC. That is, we posit that PN + BUP will have superior OUD treatment entry compared to the PN and UC arms, and that entry and retention in OUD treatment will lead to reduced opioid use and related harms, improve HIV/STI treatment adherence, and reduce HIV/STI related behavioral risks.

### Inclusion/Exclusion Criteria

Inclusion criteria are: (1) age 18 or older; (2) illicit opioid use in the past 30 days; (3) meet current DSM-5 criteria for OUD; (4) and be willing and able to provide informed consent in English. Potential participants are assessed for OUD by a research assistant (RA) using a modified version of the World Mental Health Composite International Diagnostic Interview (CIDI; [37]) that maps to DSM-5 diagnostic criteria for OUD. Research staff are trained to administer the modified CIDI for OUD as part of eligibility screening.

Exclusion criteria were: (1) current enrollment in SUD treatment for opioid use with medication (e.g., buprenorphine, methadone, naltrexone); (2) clinical contraindication with buprenorphine (e.g., allergic reactions to buprenorphine); (3) regular use of illicit long-acting opioid agonists (e.g., methadone, due to potential challenges with dose induction); (4) heavy alcohol use that raises a safety concern that precludes eligibility for buprenorphine induction (as determined by a clinician); (5) high dose or intravenous benzodiazepine use; (6) pregnancy; (7) unstable medical or psychiatric illness; and (8) inability to provide informed consent.

All eligibility screenings are reviewed by one of the study investigators for approval prior to completion of the baseline interview.

### Recruitment

Individuals with untreated OUD are recruited using a combination of strategies as described below.

#### In-clinic recruitment

The original recruitment strategy for the study involved systematic screening of patients seeking services at two public sexual health clinics. Sexual health clinic patients are informed about a health study taking place at the sexual health clinic and invited to meet with the RA to be privately screened for eligibility. This screening occurs after the clinic visit or during times when patients are waiting for clinical services. The RA obtains verbal consent for the screening, which maintains anonymity. Screening data are linked with the participant’s research record only if they are enrolled in the study and provide written informed consent.

#### Proactive invitations through telehealth

Following the need to halt in-person recruitment due to COVID-19, we adapted our protocol at allow for proactive invitations to screen for study eligibility to patients receiving telehealth services from the sexual health clinics and who consented to being contacted for research opportunities. Proactive invitations to telehealth patients were the primary recruitment strategy from July 2021 through June 2022. RAs called or texted clinic patients who agreed to be contacted to invite them to be screened for a health study. The RA obtained verbal consent for the anonymous screening and conducted the initial screening by phone. If the patient screened eligible and expressed interest in participation, RAs scheduled an in-person baseline at the research office to complete enrollment. This method was discontinued in June 2022 as telehealth appointments declined.

#### Advertisements and referrals

Recruitment flyers are posted at local health department locations and patients who are interested can contact research staff. Flyers include a link for web-based, anonymous, self-administered pre-screening using the Tobacco, Alcohol, Prescription medication, and other Substance use (TAPS) Tool [38]. The TAPS Tool is a two-part tool that consists of a 4-item screening tool for tobacco use, alcohol use, prescription medication misuse, and illicit substance use in the past year, and a brief assessment. The TAPS Tool can be self-administered or administered by an interviewer. Individuals who are provisionally eligible based on TAPS Tool questions related to opioid use and who are interested in participation are asked to provide contact information and consent to be contacted by RAs to complete the full eligibility screening. This is an ongoing recruitment method since the commencement of the study.

#### Participant referrals

After extended challenges with clinic-based recruitment, the protocol was modified and approved to expand recruitment beyond the sexual health clinics. The study was approved to allow enrolled participants to refer social network members for study screening via snowball sampling. Participants who make social network referrals are compensated for each successful referral (i.e., a referral bonus totaling $40) and may be compensated for up to five individuals who are enrolled into the study (maximum referral bonus totaling $200). To receive the referral bonus, the referred social network member must contact research staff directly, meet eligibility criteria, and complete enrollment into the study. This referral method began in May 2023.

The first wave of sampling seeds included participants who were originally recruited into the study through the sexual health clinics, reconsented on the new referral opportunity and were invited to refer peers to the study. Research staff track the source of each referral by asking potentially eligible participants how and by whom they were referred to the study. Considering ethical practice of research, anyone who is referred by a seed is instructed to contact the study team on their own volition. That is, we do not collect contact information for anyone referred by a seed, rather, the referee voluntarily contacts the research team for eligibility screening.

#### Community outreach

The protocol modifications in response to recruitment challenges also allow research staff to conduct outreach visits in the community to screen community members with untreated OUD who may be interested in study participation. Research staff inform potentially interested community members about a health study taking place and invite them to be screened anonymously for eligibility. Individuals who meet eligibility criteria are invited to participate in the study. Community outreach is defined as two approaches: passive and active. Passive community outreach involves introductions to community-based organizations that may provide (non-treatment) services to people who use drugs, for example, syringe exchange programs and harm reduction organizations. Active community outreach involves direct interactions with individuals at targeted locations near such community-based organizations, libraries, or other areas that are frequented by community members.

### Informed Consent

During the baseline interview, the RA describes the study, reviews the IRB-approved informed consent form, and explains the risks and benefits of participation. To assess understanding, RAs administer a brief consent quiz on which individuals must receive a perfect score within three attempts to be deemed eligible.

### Randomization, and Baseline Procedures

After the individual provides written informed consent, the RA administers the baseline assessments. Upon completion of all baseline assessments, the RA completes the randomization assignment and informs the participant of their study condition. Participants are assigned to conditions using a random permutation procedure and block sizes of 3, 6, and 9. RAs are blinded to randomization assignment during the administration of the baseline assessments. Participants receive compensation for the baseline assessment following randomization, in addition to receiving compensation ($40) for completion of each of the two follow-up assessments at 3- and 6-months.

### Study Conditions

#### Usual care (UC)

Participants assigned to the UC condition receive standard care from their existing care providers as appropriate. As an enhancement to Usual Care, RAs provide a study-approved list of community-based resources for substance use, mental, and sexual health care. Additionally, our research organization is a state-certified overdose response program and is equipped to provide overdose education (e.g., review of signs/symptoms of an opioid overdose and instructional education on the use of naloxone to reverse overdose events) to research participants. Following this overdose education, participants who are interested, are at risk of overdose, or who encounter others at risk, are provided naloxone at no cost.

#### Patient navigation services (PN)

Participants assigned to the PN condition receive the same standard care from their providers described for UC. In addition, PN participants meet with a Patient Navigator immediately following randomization at the baseline visit. During the initial meeting, the Patient Navigator assesses readiness for SUD treatment, barriers to care, and other social determinants of health. The Patient Navigator delivers a motivational intervention as appropriate, develops rapport, and makes a treatment plan with the participant. Using patient navigation and motivational intervention techniques, the Patient Navigator continues to coordinate with participants for up to 2 months to identify available resources and strategies to resolve discussed barriers (e.g., transportation, insurance coverage, governmental assistance). Navigators have a small fund available (typically not exceeding $100 total per participant) to assist patients with needs such as medical co-pays, obtaining ID cards, transportation, low-cost phones/cell phone minutes, and other related items. Table 1 shows examples of common barriers and how the Navigators would address them, as adapted from our team’s previous patient navigation work [15,39].

During the start-up phase of the study, Patient Navigators familiarized themselves with the available treatment modalities and providers in the local SUD and healthcare systems by visiting various programs and creating connections with program intake coordinators and clinical teams in the community. For example, Navigators created a database of community-based programs containing details on requirements of each program to inform their referral process based on individual circumstances. Additionally, prior to the start of the study, the Navigators completed motivational interviewing training offered by the Patient Navigator Training Collaborative and through the state’s certified peer recovery specialist program. Fidelity to the patient navigation intervention will vary depending on the level of perceived need and willingness to engage with the navigator, thus fidelity will be tracked across different levels. At minimum, fidelity to the patient navigation intervention will be considered as completion of the intake session following randomization to one of the patient navigation arms. During this session, the navigator completes a needs assessment with the individual and provides an overview of the types of services offered. Fidelity will also be measured by examining the level of engagement with the patient navigator, including the number of service encounters within the 2-month timeframe of intervention delivery.

#### Patient navigation + buprenorphine initiation (PN + BUP)

Participants randomized to the PN+BUP Arm will meet with a provider who can prescribe buprenorphine and the Patient Navigator (typically together) following randomization. The initial buprenorphine prescribing visit can occur in person or remotely via telehealth. The same types of services will be provided by the Patient Navigator as in the PN Arm.

In addition, participants in the PN + BUP Arm will be assessed for medical appropriateness and be provided clinical directions for an unobserved, at-home initiation of buprenorphine via an approximately seven-day prescription. Following this initial bridge prescription from an OUTLAST-B affiliated provider, the participant and the Patient Navigator will coordinate to facilitate the participant’s transfer to a buprenorphine provider in the community. If unforeseen delays in linkage to community treatment occur, there is an opportunity to be re-assessed by the study clinician (MT) to receive an additional bridge prescription while the Patient Navigator continues to work with the participant towards their community-based linkage. Following linkage to another provider, the Patient Navigator continues to work with the participant to support their early engagement in OUD treatment.

##### Buprenorphine Initiation.

The study uses the buprenorphine/naloxone (bup/nal) combination product available through local pharmacies. Participants are assessed by the provider and given initiation directions based on participant preference and prior experiences using buprenorphine. For example, participants could be instructed to take their first dose of 4/1 mg bup/nal sublingually once they begin to feel symptoms of opioid withdrawal and to take an additional 4/1 mg (if needed) 1–2 h following their first dose. Participants can take an additional 4/1 mg (if needed) 2–3 h after the second dose, for a maximum first day dose of 12/3 mg. On the second day, they could be instructed to take 8/2 mg in the morning, with an additional dose up to 8/2 mg if needed (up to 16/4 mg of bup/nal daily). The prescribing provider will have flexibility to start participants at a different initial starting dose (e.g., 2/0.5 mg bup/nal based on microdosing protocols) if the provider and participant are concerned about precipitated withdrawal. The Patient Navigator will use initial contacts with the participant to assess for any events of precipitated withdrawal following buprenorphine initiation within the first few days following the receipt of the medication (defined as 1–3 days after the receipt of the prescription). In addition, participants are encouraged to contact study staff if they have any additional questions or concerns.

### Assessments

Assessments were conducted by trained RAs as outlined in Table 2. The RAs are blinded to study condition at baseline (i.e., assessments are administered prior to randomization). RAs are not blind to study condition at follow-up visits. All participants are sought for follow-up assessments at 3- and 6-months post-enrollment. Assessments consist of a battery of instruments to measure outcomes including, by not limited to: OUD severity (modified World Mental Health Composite International Diagnostic Interview [CIDI]) [37], substance use (Addiction Severity Index-Lite) [40], health and functioning (World Health Organization Quality of Life [WHOQOL-BREF]) [41], psychological stress (Kessler-6 scale) [42], and treatment utilization data. Urine toxicology is collected at baseline, 3-, and 6-month follow-up interviews and tested for fentanyl, opiates, oxycodone, methadone, buprenorphine, cocaine, cannabis, alcohol, amphetamines, and benzodiazepines. Following the increase of xylazine in the illicit drug supply, an additional dip test for xylazine was included in the urine toxicology panel.

### Primary Outcome

The primary outcome is OUD treatment entry within 30-days post-enrollment, defined as admission to a buprenorphine provider (either office-based care or a specialty OUD program) or alternative OUD treatment modalities (e.g., methadone, medical detox followed by behavioral treatment). OUD treatment entry and retention will be collected by self-report. To the extent possible, treatment data will be verified via provider or treatment records with participants’ written permission. Treatment retention (defined as total number of days in MOUD treatment) will be examined as a secondary outcome. OUD treatment entry within 30-days of study enrollment is the primary outcome because linkage to such treatment is the main and most proximate goal of the service strategies being tested.

To assess for treatment entry and retention, at each follow-up assessment, participants are asked whether they enrolled in treatment for drugs or alcohol since their last assessment. If the participant reports one or more treatment episodes, research staff collect details of each episode including start/end dates, type of setting (e.g., inpatient, outpatient, detox), receipt of medication (e.g., buprenorphine, methadone), and whether they attended self-help groups.

### Statistical Analysis

Outcomes will be examined via a generalized linear modeling framework (for endpoint analyses, such as OUD treatment entry), with extension to generalized linear mixed modeling for repeated measures models (for analyses of differential change, e.g., days of drug use).

#### Outcome variables

Outcome variables will be either: (1) dichotomous variables (e.g., entry into treatment, urine test results), assumed to follow a binomial distribution; or (2) discrete random variables (e.g., days of drug use), assumed to follow a Poisson distribution. All distributional assumptions will be evaluated prior to analyses, and if violated, suitable alternatives will be chosen (e.g., allowing for over-dispersion in Poisson models).

#### Explanatory variables

The explanatory variables in the statistical model will include: (1) *Study Condition* (*PN + BUP vs PN vs Usual Care)* and (2) *Recruitment Site (Clinic Site, Social Network, Other)*. For variables analyzed as repeated measures, the model will also include (3) *Time* and its interaction with *Condition*.

#### Hypothesis tests

For each outcome model, an omnibus test of the Condition factor will test whether the outcome differs across study arms. Single-degree-of-freedom contrasts will test the specific hypotheses that PN+BUP is superior to PN, which in turn is superior to UC. The contrasts correspond to the Condition effect for endpoint analyses (e.g., treatment entry; early remission of DSM-5 OUD) or the Condition X Time interaction for repeated measures analysis of differential change (e.g., Quality of Life).

### Sample Size and Power

The study originally targeted recruitment of 360 participants. However, the COVID-19 pandemic led to significant challenges in recruitment. Efforts have increased substantially since the introduction of social network referrals; however, alternative design strategies may be considered because of low enrollment rates (e.g., adjustment to targeted sample size or collapsing groups).

## PROGRESS TO DATE AND FUTURE DIRECTIONS

After a delayed start-up due to the COVID-19 pandemic and largely unsuccessful adaptations to recruit patients seen via telehealth, in-person recruitment at the two sexual health clinic sites commenced in November 2021. While research staff were able to successfully integrate into the clinic flow and screen a large number of patients for eligibility (>4000 patients), new challenges that continued to hinder study enrollment became apparent.

A significant challenge impacting recruitment has been the sustained reduction in patient volume, even as the clinics resumed in-person services following the COVID-19 pandemic. Services were limited to scheduled appointments only, which differs from the pre-pandemic model of high-volume, on-demand walk-in services. This has resulted in both fewer patients served at the sexual health clinics overall, and a different patient population than originally anticipated. Specifically, the rate of untreated OUD (the core inclusion criterion for the study) is lower than expected, based on preliminary data obtained in the feasibility pilot [11]. As of February 2023, walk-in services at the clinics have resumed, but rates of untreated OUD have remained low (see Table 3). Based on screening data for patients seeking sexual health services through July 2023 using the TAPS Tool and branching to questions about past 30-day opioid use, only 2.4% have endorsed any opioid use in the 30-days prior to screening. Eligibility rates were so low that the viability of the study was in question.

In response to low rates of eligibility and enrollment, we modified the protocol to expand recruitment beyond the sexual health clinic sites, while maintaining the originally planned screening activities at these sites. There has been some promise with in-person screening efforts because walk-in services have resumed, though not to the volume observed prior to the pandemic and corresponding shifts in clinical flow. As described above, recruitment procedures were adapted for recruitment using snowball sampling (i.e., social network member referrals from enrolled participants) and engagement with other healthcare and community sites to increase overall recruitment efforts.

The social network referral strategy has singularly improved recruitment efforts compared to the clinic screening. Between May 2023 and July 2023, 79 participants were enrolled—a significant increase in enrollment compared to those in 2021 and 2022. Using this novel recruitment strategy, we may be able to understand if Patient Navigation techniques can not only improve linkages to OUD treatment among sexual health patients, but also facilitate service linkage for those referred by their peers. Notably, this strategy has some parallels with how sexual health clinics already function in another domain, namely through disease intervention specialists that identify and treat partners of individuals with sexually transmitted infections. Evaluating these touchpoints as facilitators to OUD treatment may shed light on how researchers and clinicians can engage individuals with untreated OUD using novel strategies such as social network connection. These techniques may resolve barriers to engagement in research such as institutional distrust [43] by allowing potentially eligible participants to learn about research opportunities through their trusted peers. Furthermore, it is important to examine how social networks may be a viable pathway to encourage and empower individuals with untreated OUD to seek evidence-based treatment services that they otherwise would not pursue independently. Still, there are potential confounders that may be introduced through the use of social network referrals. Social support has been identified as a predictor of treatment entry and engagement, but findings are mixed [44-46]. It is possible that greater social network support may influence readiness for treatment entry. Thus, it will be important to consider the association of social networks, such as familial support, on the primary outcome of treatment entry in this study.

## LIMITATIONS

There are potential limitations to the OUTLAST-B study that should be noted. With respect to the outcome of measuring incidence of new sexually transmitted infections, we are limited by the length of follow-up. Follow-up research interviews are conducted at 3- and 6-months after the baseline assessment, which may not allow for an adequate follow-up time for new incidence of STIs. We are passively tracking health outcomes through administrative data through 12-months of follow-up, which may mitigate some of these concerns, however, it is possible that 12-months of follow-up is too short to properly detect this outcome. There are also limitations associated with the use of social network referrals. Because “successful” referrals are incentivized, there is a potential that those making referrals have informed their peers about requirements of study eligibility and that those referred to the study do not have OUD. Though there is no way to identify these potential occurrences with complete accuracy, research staff are trained to be diligent about responses to study eligibility criteria and throughout the baseline assessment. Deviations or inconsistencies in self-report related to substance use and treatment history are closely monitored and reviewed by study investigators and the study clinician.

## CONCLUSIONS

The OUTLAST-B study weathered considerable recruitment challenges stemming from the COVID-19 pandemic. Recent adaptations to broaden recruitment have seen early success while maintaining the core focus and aims of the study. Despite the challenges described, the study has the potential to inform the field about the effectiveness of two treatment linkage strategies (Patient Navigation, with and without rapid a buprenorphine bridge) for people with untreated OUD.

## Figures and Tables

**Table 1.** Examples of Potential Barriers to Engaging in Care and Navigator Response.

Example of Barrier	Navigator response
Ambivalence for SUD treatment	Use motivational interventions to explore and resolve ambivalence; Deliver basic education to address health beliefs and increase health literacy about treatment options.
Discomfort interacting with treatment staff	Explore underlying reasons for patient discomfort (e.g., low health literacy; perceived stigma) and address with education and/or role playing; Advocate for patient with treatment staff.
Lacks health insurance or has insufficient coverage	Identify appropriate insurance eligibility and options; Help patient fill out application and interface with insurance bureaucracies on patient’s behalf.
Cannot afford recommended medicines	Identify and help patient sign up for prescription assistance programs; Interface with physician(s) to discuss less costly alternatives.
SUD treatment program requires photo ID	Identify nearest DMV; Assist with transportation; Assist with fees.
Recommended care is far or inconvenient	Assist with transportation; Facilitate transfer to closer providers.
Missed appointment	Appointment reminders; Accompany patient to appointment; Reschedule.

Note: This table has been adapted from previously published patient navigation work from our team [39].

**Table 2.** Data collection schedule and measures.

Measures	Baseline	3-month	6-month
Illicit drug use (urine test)	♦	♦	♦
Substance use patterns (ASI-Lite)	♦	♦	♦
OUD diagnostic criteria (Modified CIDI)	♦	♦	♦
HIV risk behavior	♦	♦	♦
Quality of Life (WHOQOL-BREF)	♦	♦	♦
Psychological distress (Kessler-6)	♦	♦	♦
Patient navigation satisfaction		♦	
SUD treatment utilization	♦	♦	♦

**Table 3.** Screening rates by strategy from 2021 through July 2023.

Screening Metric	Single-Item Screening		TAPS Tool In-Person	Network Referrals
	Telehealth	In-Person		
Total records	108	810	4059	136
Completed brief OPI screen	107 (99.1)	802 (99.0)	3874 (95.4)	133 (97.8)
Past 30-day opioid use	11 (10.1)	36 (4.4)	98 (2.4)	128 (94.1)
No past 30-day opioid use	96 (88.9)	766 (94.6)	3776 (93.0)	5 (3.7)
Missing	1 (0.9)	8 (1.0)	185 (4.6)	2 (2.2)
Completed TAPS Tool screen	N/A	N/A	2429 (65.8)	N/A
Missing			1265 (34.2)	

Notes: TAPS Tool = Tobacco, Alcohol, and other Substance use Tool.

## Data Availability

The dataset of the study is available from the authors upon reasonable request.
